# The Tonic Treatment of Syphilis

**Published:** 1877-05

**Authors:** 


*The Tonic Treatment of Syphilis.* By E. L. KEYES, A. M., M. D. Adjunct Professor of Surgery, and Professor of Dermatology in the Bellevue Hospital Medical College, etc. pp. 83, 8vo. New York: D. Appleton & Co.

The studies of the author upon the effect of long continued small doses of mercury in increasing the abundance of the red corpuscles of the blood, in syphilis, as determined by actual countings with the microscope, and in exerting a general tonic influence upon the system, were made public in an article in the *American Journal of Medical Sciences*, something more than a year ago. A paper presented at the Section of Dermatology and Syphilis, at the recent International Medical Congress, embodied the same researches. The profession of tbe country is doubtless very generally aware of the results of our author's experiments in blood counting, and are ready to welcome the brief and very readable exposition he now gives of the treatment of syphilis, which his study and extended observation recommends, and which conforms closely in the main to that which modern practice has adopted.

The first scientific effort to determine the effect of syphilis, and the use of mercury and the iodide of potassium in this disease, upon the blood, was by Grassi in 1844: Ricord subsequently, Liegeois is 1869, and Wilbouchewitch in 1874, published results of experiments which tended to show that mercury at first increased, and with continued use diminished the abundance of red corpuscles of the blood. Keyes observes that in the experiments which showed a diminution of red corpuscles, the size of the dose used was such as to produce diarrhœa, with general irritation and loss of weight, while from his own carefully conducted experiments, he concludes that by the long continued use of small amounts of mercury, such as the patient can bear without intestinal or other irritation, a general tonic effect is produced upon the system and syphilitic manifestations are in a very satisfactory degree controlled.

He concludes that the average number of red corpuscles in the blood of a person in health is from four and a half to five millions in a cubic millimeter, that anemia and syphilis reduce their number, that mercury or iodides in small well regulated doses act as a tonic, increasing their abundance and controlling as is generally accepted, syphilitic disease. The dose he has found can be suitably continued for long periods, varies from a half to two grains daily of the proto-iodide, in three parts, or one forty-eight to one-twelfth a grain of the biniodide or bichloride of mercury, thrice daily. When active treatment of syphilitic manifestations is desired, the size of the dose is just sufficient to produce slight symptoms of the peculiar effect of mercury or iodine in the particular individual; the quantity thus determined is termed the "full dose," and after manifestation of syphilis has subsided, half that amount, termed the "reserve dose," is continued for a long period, two and one-half or three years, or more in all.

The undesirable effects of full doses of mercury or of iodism are well described. The method of inunction, mercurial vapor baths, the method with infantile syphilis and the duration of treatment are fully set forth as also the treatment of the various local lesions of the disease. Those who are interested in being thoroughly informed as to modern practice in the treatment of syphilis will find pleasure and profit in reading the work. W.

